# Genetic Prediction of Eye, Hair, and Skin Color: Forensic Applications and Challenges in Latin American Populations

**DOI:** 10.3390/genes16101227

**Published:** 2025-10-16

**Authors:** Beatriz Armida Flores-López, Anna Guadalupe López-Ceballos, Cristal Azucena López-Aguilar, Manuel Alejandro Rico-Méndez, Kesia Lyvier Acosta-Ramírez, Alan Cano-Ravell, Gildardo Gembe-Olivarez, Andres López-Quintero, José Alonso Aguilar-Velázquez, Jorge Adrian Ramírez-de-Arellano Sánchez, José Miguel Moreno-Ortiz

**Affiliations:** 1 Departamento de Ciclo de Vida, Facultad de Medicina, Universidad Autónoma de Guadalajara, Zapopan 45129, Jalisco, Mexico; beatriz.flores@edu.uag.mx; 2 Instituto de Genética Humana “Dr. Enrique Corona Rivera”, Departamento de Biología Molecular y Genómica, Centro Universitario de Ciencias de la Salud, Universidad de Guadalajara, Guadalajara 44340, Jalisco, Mexico; anna.lopez4758@alumnos.udg.mx (A.G.L.-C.); cristal.lopez2278@alumnos.udg.mx (C.A.L.-A.); manuel.rico8557@alumnos.udg.mx (M.A.R.-M.); acostakesia@gmail.com (K.L.A.-R.); alan.cano1006@alumnos.udg.mx (A.C.-R.); 3 Maestría en Genética Forense e Identificación Humana, Centro Universitario de Ciencias de la Salud, Universidad de Guadalajara, Guadalajara 44340, Jalisco, Mexico; 4 Instituto de Nutrigenética y Nutrigenómica Traslacional (INNUGET), Departamento de Biología Molecular y Genómica, Centro Universitario de Ciencias de la Salud, Universidad de Guadalajara, Guadalajara 44340, Jalisco, Mexico; gildardo.gembe7818@alumnos.udg.mx (G.G.-O.); andres.lopezq@academicos.udg.mx (A.L.-Q.); 5 Laboratorio de Ciencias Mofológico Forenses y Medicina Molecular, Departamento de Morfología, Centro Universitario de Ciencias de la Salud, Universidad de Guadalajara, Guadalajara 44340, Jalisco, Mexico; josealonso.aguilarvelazquez@academicos.udg.mx; 6 Laboratorio de Investigación en Cáncer e Infecciones, Departamento de Biología Molecular y Genómica, Centro Universitario de Ciencias de la Salud, Universidad de Guadalajara, Guadalajara 44340, Jalisco, Mexico

**Keywords:** forensic DNA phenotyping, human identification, externally visible traits, genetic prediction, forensics

## Abstract

Forensic DNA phenotyping (FDP) is an important innovation approach in forensics sciences, especially when traditional DNA profiling results are limited, mostly due to the absence of reference samples. FDP is based on the detection of genetic variants in specific genes whose function is related to pigmentation mechanisms and uses the genotypes found in the sample to determine the externally visible traits (EVT) such as the iris, hair, and skin tone or color of the individual; this prediction would help and expedite human identification processes and solve criminal cases. Several technologies have been developed to facilitate EVT prediction; however, most of them have been validated only in European populations. Implementing techniques for FDP in Latin American countries is essential given the problems of disappearance and human identification that have persisted for years. Nonetheless, scientists have a great challenge due to the admixed genetic structure of the population. This review explores the current application of FDP, emphasizing its significance, practical uses, and limitations within Latin American populations.

## 1. Introduction

Human identification is a challenge in forensic science, especially in cases involving severely decomposed, fragmented, or fully skeletonized remains [1,2]. Traditional forensic DNA profiling based on short tandem repeats (STRs) is effective for individual identification by comparison with reference samples [3]. However, its applicability is limited when reference samples or biological relatives are unavailable. In such cases, the prediction of externally visible traits (EVTs) from DNA analysis has emerged as a valuable complementary tool to refine investigative leads [4,5,6].

Forensic DNA phenotyping (FDP) is a genetic approach aimed at inferring externally visible traits by analyzing specific single nucleotide variants (SNVs) associated with pigmentation pathways [7,8]. This method has shown substantial potential in accurately predicting key external traits, thereby aiding the generation of genetic composite images or supporting witness-based reconstructions. A wide range of features has been explored for their potential use in FDP, including predicting pigmentation of iris, hair, and skin [9]; determining eyebrow color [10]; identifying the presence of freckles [11]; assessing hair shape [12]; detecting male pattern baldness [13]; and estimating body height [14]. However, among these traits, eye, hair, and skin color have been the most extensively researched and validated, owing to their genetic determinism and relative phenotypic stability [5,8,15].

In Latin America, the implementation of FDP is at an emerging stage [16]. Nonetheless, its potential significance is substantial, particularly in regions affected by armed conflicts, organized crime, and forced disappearances. The genetic diversity and admixture typical of Latin American populations arising from Indigenous, European, and African ancestries represent both a distinctive opportunity and a methodological challenge for accurate phenotypic prediction [17,18,19].

This review examines the genetic prediction of eye, hair, and skin pigmentation in forensic contexts, highlighting its relevance, practical applications, and inherent limitations within Latin American populations. By examining the current research landscape, predictive models, and region-specific factors, it seeks to contribute to the advancement and ethically responsible implementation of this emerging forensic tool across Latin American populations.

## 2. Genetic Basis of Eye, Hair, and Skin Color

**Figure 1:**
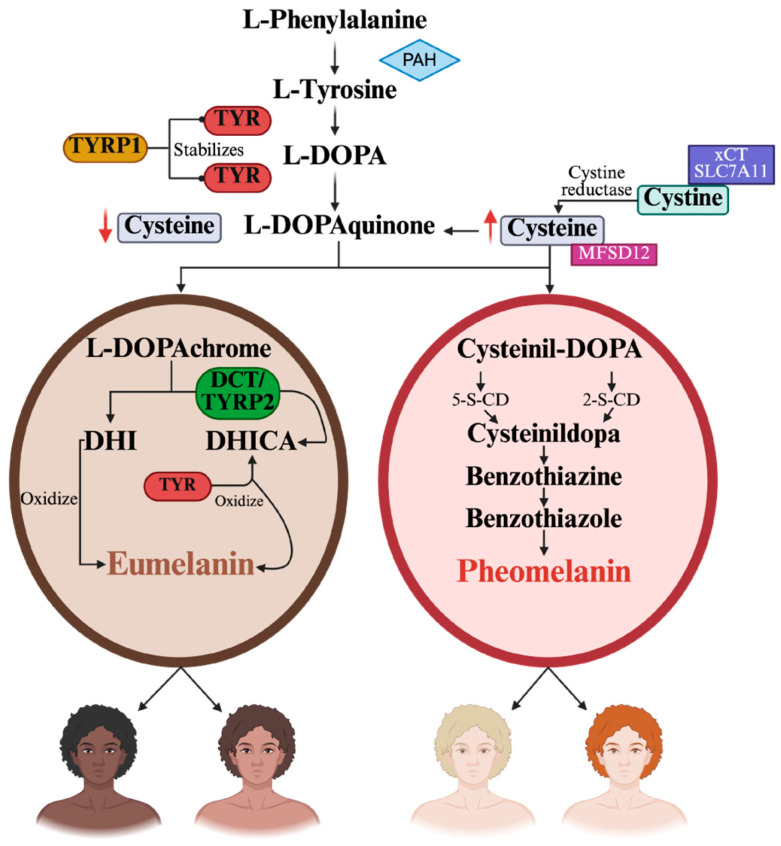
**Schematic representation of melanogenesis in humans and the biogenesis of eumelanin and pheomelanin.** L-phenylalanine is converted to L-tyrosine and oxidized by TYR to form L-dopaquinone, the branching point for eumelanin and pheomelanin synthesis. Under low-cysteine conditions, dopaquinone is transformed into dopachrome and then polymerized into eumelanin (brown/black pigment). Under high-cysteine conditions, it conjugates with cysteine to produce cysteinyldopa intermediates that polymerize into pheomelanin (yellow/red pigment). Created in BioRender. Gyc, L. (2025) https://BioRender.com (accessed on 12 October 2025).

Human pigmentation traits, including eye, hair, and skin color, are complex phenotypic characteristics influenced by the interaction of multiple genes that regulate the type, quantity, and distribution of melanin synthesized by melanocytes [20]. The two primary forms of melanin are eumelanin (brown/black pigment) and pheomelanin (red/yellow pigment), contributing to the wide range of pigmentation seen in human populations (Figure 1) [21]. Melanin synthesis begins in the cytosol of melanocytes, where L-phenylalanine is converted into L-tyrosine by phenylalanine hydroxylase (PAH). Tyrosine, together with L-3,4-dihydroxyphenylalanine (L-DOPA), is oxidized by tyrosinase (TYR) at the melanosomal membrane, leading to the formation of L-dopaquinone. Tyrosinase-related protein 1 (TYRP1) stabilizes TYR and facilitates its translocation to the melanosome. Dopaquinone constitutes the branching point for the synthesis of the two major types of melanin: eumelanin and pheomelanin. Under conditions of low cysteine availability, dopaquinone is converted to dopachrome, which, through the action of dopachrome tautomerase (DCT) and tyrosinase-related protein 2 (TYRP2) (DCT/TYRP2), generates DHICA (5,6-dihydroxyindole-2-carboxylic acid), or alternatively undergoes spontaneous decarboxylation to yield DHI (5,6-dihydroxyindole). DHICA undergoes oxidative polymerization mediated by TYR, while DHI is also oxidized; both polymerize to form eumelanin, responsible for brown and black pigmentation. Conversely, under high cysteine availability, resulting from the reduction of imported cystine (via the xCT/SLC7A11 transporter) by cystine reductase, cysteine is transported into the melanosome by MFSD12. Dopaquinone conjugates with cysteine to generate cysteinyldopa, which undergoes oxidation and cyclization to produce benzothiazine and benzothiazole intermediates. These intermediates polymerize into pheomelanin, which accounts for yellow to reddish pigmentation [22,23,24].

Genetic studies have identified several key genes and variants associated with pigmentation, many of which hold significant relevance for forensic phenotyping [25]. Unlike most genetic traits, pigmentation shows large differences among continental populations due to strong natural selection along latitudinal gradients [26].

For eye color, two major genes play a central role: *HERC2* and *OCA2*, both located on chromosome 15. The intronic SNV rs12913832 in *HERC2* is the primary determinant of eye color, modulating *OCA2* expression through chromatin loop formation [27,28,29]. While rs12913832 is strongly associated with blue and brown eye colors, additional variants in the OCA2-HERC2 region can explain unexpected blue eyes in individuals with brown eye genotypes. When this SNV is present in its derived form, it reduces *OCA2* activity, resulting in lower melanin levels in the iris and producing blue eye color. Conversely, the ancestral form of the SNV leads to higher *OCA2* expression and the production of brown eyes [30,31].

In the case of hair color, genes such as *MC1R*, *TYR*, and *SLC45A2* are critical. The *MC1R* gene is strongly associated with red hair and fair skin. Variants in *MC1R* reduce its activity, leading to increased pheomelanin and a red hair phenotype. For example, D84E, R151C, R160W, and D294H variants show significantly reduced functional activity and are strongly linked to the red hair color (RHC) phenotype [32]. On the other hand, *TYR* encodes the tyrosinase enzyme, which is essential for melanin synthesis, and *SLC45A2*, which influences melanosome function, contributing to lighter or darker hair shades depending on the presence of specific alleles [20,33].

Genome-wide association studies have expanded our understanding of the genetic basis of human skin pigmentation. Multiple genes have been identified as key contributors, including *SLC24A5*, *SLC45A2*, *TYR*, *TYRP1*, *OCA2*, *HERC2*, *MC1R*, and *ASIP* [34,35,36]. Among these, *SLC24A5* has been identified as a major determinant of lighter skin. The SNV rs1426654 in this gene, which results in an alanine to threonine substitution, is strongly associated with reduced melanin content in the skin, resulting in pale skin [37,38]. Similarly, *SLC45A2* and *TYR* affect melanin production and melanosomal activity, while *HERC2*, particularly the SNV rs12913832, regulates *OCA2* expression through long-range enhancer interactions, influencing eye and skin color [25,28]. *OCA2* itself is independently associated with eye color [25].

Human pigmentation involves complex interactions between multiple genes and regulatory elements [39], but, considering epistatic effects can improve prediction accuracy for some pigmentation traits [40]; for example, MC1R interacts with HERC2 and VDR to influence hair and skin color [25,40]. *SLC24A5*, *TYR*, and *SLC45A2* are other major loci affecting skin color [41]. Gene–gene interactions contribute significantly to eye color variation, including *HERC2-OCA2* for hazel eyes and *HERC2-SLC24A4* for blue eyes [42].

Studies conducted in Latin American populations have provided valuable insights into the genetic architecture of pigmentation in highly admixed groups. Adhikari et al. (2019) reported 18 significant association signals distributed across 12 genomic regions in a cohort of over 6000 individuals, identifying novel loci and a major *MFSD12* variant commonly found in populations with East Asian and Native American ancestry [43]. Therefore, the relationship between genetic ancestry and skin pigmentation varies notably across the region, displaying correlations that range from strong to weak [44].

## 3. Phenotype Prediction Tools

FDP tools are designed to infer EVTs such as eye, hair, and skin color from genetic data. These tools are particularly valuable in forensic investigations where conventional identification methods are unavailable or ineffective. The first system developed for predicting EVTs was IrisPlex, which reported the conditions necessary for predicting eye color. It is based on six SNVs in key genes such as *HERC2* and *OCA2*, with high accuracy in distinguishing between blue, intermediate (green and hazel tones), and brown eyes, with prediction rates exceeding 90% in European populations. Subsequently, HIrisPlex was developed, which allows the inference of the color of eyes and hair, incorporating 22 additional SNVs in genes such as *MC1R*, OCA2, *SLC24A4*, *SLC24A5*, *SLC45A2*, and *TYR*, enabling the prediction of hair color (black, brown, blond, and red) with a reliability of over 75%. More recently, the HIrisPlex-S system was created (https://hirisplex.erasmusmc.nl/), which is based on 41 SNPs for predicting eye, hair, and skin color [6,15]. The system originated and has been validated in European populations, and has demonstrated high predictive accuracy for certain traits, particularly blue and brown eyes (~90%), and to a lesser extent, hair and skin color. Predictions are based on a Bayesian classifier that estimates the likelihood of each phenotype category based on the individual’s genotype [45].

Other models beyond HIrisPlex-S have explored the use of allele frequency-based approaches and decision tree algorithms [45]. Frequency-based models estimate phenotype probabilities by comparing an individual’s genetic variants with known allele distributions across populations [46]. Decision trees and other machine learning methods use large datasets of known genotype-phenotype pairs to identify decision rules that classify individuals into phenotype categories based on their SNV profiles [47]. These approaches can account for gene–gene interactions and have shown promise in improving the classification of intermediate phenotypes, although they require extensive, well-curated datasets for training and validation [48].

Despite these advances, there are still significant limitations when applying these tools to non-European or admixed populations, such as those in Latin America, South Asia, or Sub-Saharan Africa [49]. The predictive accuracy tends to decrease in these populations due to differences in allele frequencies, the presence of population-specific variants not included in the models, and the complex patterns of genetic admixture [50]. As a result, predictions in admixed individuals may be less reliable or more ambiguous, particularly for traits like skin tone, which exhibit greater variability across ancestries [51].

Ongoing efforts aim to expand reference datasets to include greater global diversity and to develop population-specific prediction models [52]. It remains crucial for forensic practitioners to interpret FDP results within the appropriate genetic and demographic context, and to communicate the probabilistic nature and inherent limitations of phenotype prediction when used in real world forensic scenarios [53].

Genome-wide association studies (GWASs) have advanced phenotype prediction by identifying common and rare variants that explain a substantial proportion of trait variability. The largest GWAS on human eye color identified 50 novel loci and confirmed 11 known genes, accounting for over half of the phenotypic variance in Europeans and addressing much of the previously missing heritability. Several loci also affect eye color in Asians, indicating shared genetic mechanisms across ancestries. The inclusion of European and non-European cohorts revealed both overlapping and distinct architectures among pigmentation traits: while many loci influence eye, hair, and skin color, others such as *TRAF3IP1* and *SEMA3A* appear specific to eye pigmentation and may act through iris structural variation rather than melanin metabolism [54].

Integrating multi-ancestry GWAS data improves the transferability of genetic signals and reduces biases from single-ancestry models. Incorporating allele frequency variation and local ancestry information increases predictive accuracy by capturing ancestry-specific effects. These advances enhance the calibration and generalization of polygenic models and support their application to forensic and anthropological phenotype prediction [54,55].

## 4. Forensic Applications in Latin America

FDP is an emerging and powerful tool with significant potential in Latin America [16], a region characterized by high biological diversity and a complex history of armed conflict, forced migration, and mass disappearances. In cases where traditional identification methods such as fingerprints, dental records, or facial recognition are unavailable, inferring physical traits like eye, hair, and skin color from DNA can provide crucial investigative leads [56,57].

A key contribution of FDP is the possibility of generating DNA-based composite sketches. These reconstructions, built solely from genetic data, provide a partial but valuable representation of an unidentified individual’s appearance [58,59]. Such predictions can guide the search process, facilitate recognition by relatives, and support cross-referencing with missing persons databases. This is particularly relevant in cases involving skeletal remains or degraded DNA, commonly encountered in clandestine graves or cold cases that have remained unresolved for decades [53,60].

The relevance of these technologies is particularly significant in Latin America, where thousands of individuals remain missing due to past and ongoing sociopolitical violence. Countries such as Mexico, Colombia, Guatemala, El Salvador and Argentina have experienced internal conflicts and state-related disappearances [61]. Additionally, the region is currently witnessing large-scale transnational migration, which has led to a new wave of disappearances, often lacking formal documentation or familial references. In these scenarios, FDP serves as a complementary tool within broader humanitarian and legal identification efforts [62].

Although still in the validation and standardization stages, several academic and forensic institutions in Latin America have begun exploring and testing phenotypic prediction tools [49]. In Mexico, for example, research groups are evaluating the SNVs included in the HIrisPlex system using massive parallel sequencing (MPS), highlighting the challenges posed by highly admixed populations and the predominance of dark phenotypes [63,64]. In Brazil, studies have assessed the accuracy of these tools and highlighted the need for regional calibration [65]. Meanwhile, forensic organizations such as the Argentine Forensic Anthropology Team (EAAF) have expressed interest in incorporating FDP into complex identification cases, particularly those related to past human rights violations [66].

The application of FDP in Latin America, is particularly challenging due to the region’s highly admixed genetic structure. Latin American populations typically exhibit a tri-hybrid ancestry composed of European (primarily Spanish and Portuguese), Indigenous, and African genetic components [17,18]. This genetic diversity results in high variability in pigmentation traits, even within the same country or community, and can reduce the predictive accuracy of existing models, which were primarily developed using European reference data [67].

**Figure 2:**
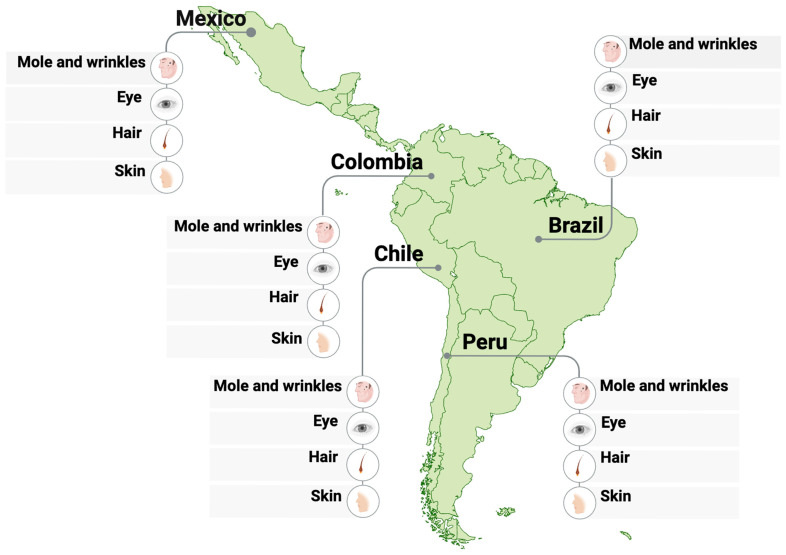
**Phenotyping studies in Latin America.** The map illustrates research carried out in Mexico, Colombia, Chile, Peru, and Brazil, focusing on EVTs such as eye, hair, and skin color, as well as mole and wrinkle patterns [43,49,63,70,71,72,73]. Created in BioRender. Gyc, L. (2025) https://BioRender.com (accessed on 12 October 2025).

To handle this, it is essential to develop prediction models based on local genomic datasets and to incorporate ancestry-informative markers that more accurately reflect the regional diversity. Without such adjustments, there is a risk of misclassification or oversimplification, particularly when attempting to infer phenotype or biogeographic origin in admixed individuals [68]. Despite these challenges, the integration of FDP into forensic science in Latin America holds great promise, especially when combined with other identification methods in multidisciplinary efforts aimed at truth, justice, and reconciliation (Figure 2) [69].

## 5. Ethical and Technical Challenges

The application of FDP, while promising, raises several ethical and technical concerns that must be carefully addressed, especially in the context of Latin America. One of the primary technical challenges is the reduced prediction accuracy in admixed individuals [74]. Current phenotype prediction models, including widely used systems such as HIrisPlex-S, were primarily developed and validated using individuals of European descent. When applied to individuals with mixed ancestries such as those common in Latin America, where European, Indigenous, and African genetic contributions are deeply interwoven, the predictive accuracy of these models often declines [65]. This can lead to incomplete or misleading phenotype estimations, which in turn may misdirect investigations or delay the identification process [75].

These technical limitations are closely linked to broader ethical concerns, particularly regarding the potential for racial profiling or the misuse of phenotypic data [76]. The use of DNA to infer traits such as skin, eye, or hair color risks reinforcing racial stereotypes or contributing to discriminatory practices if not properly contextualized [77]. For example, in law enforcement or border control settings, FDP data could be misused to justify targeting individuals based on perceived physical traits rather than objective evidence [78]. Moreover, if phenotypic predictions are interpreted without nuance, they may lead to overgeneralizations about biogeographic ancestry which are especially problematic in socially diverse societies like those in Latin America [18].

Another significant issue is the absence of clear legal frameworks governing the use of FDP in most Latin American countries. Unlike regions such as the European Union, where guidelines exist to limit the scope and use of phenotypic prediction in forensics, many Latin American nations lack comprehensive regulations or oversight mechanisms [16]. This legal vacuum creates uncertainty regarding how and when FDP can be used, who has the authority to request or interpret such data, and what protections exist for individuals whose DNA is analyzed. Without clear standards, there is a heightened risk of misuse, lack of transparency, and erosion of public trust in forensic institutions [79].

To mitigate these risks, institutional ethics committees must oversee project approval, data collection, and analysis protocols to ensure compliance with national and international regulations. Ethical principles are enforced through standardized institutional frameworks that guarantee transparency and data protection [80,81,82]. Informed consent procedures should explicitly define the study purpose, forensic applications, data-sharing limitations, storage period, and the right to withdraw participation [80]. All personal and genetic data must be anonymized and encrypted to prevent individual identification. Access to databases should follow tiered authorization, restricted to trained and accredited personnel under confidentiality agreements. All processes must remain under continuous audit by ethics committees and data protection authorities, in accordance with national and international guidelines on genetic data governance and forensic research [81]. It is important to mention that informed consent should not be treated as a formality, but as a meaningful ethical obligation that respects the rights and dignity of individuals and their families [82]. Similarly, institutions must implement strong data governance policies to ensure confidentiality, prevent unauthorized access, and protect sensitive genetic information from being exploited or leaked [83].

While FDP provides valuable genetic information for predicting externally visible traits, its application must consider specific methodological constraints such as limited predictive accuracy in highly admixed populations and the variable effect size of pigmentation associated variants. To promote reliable implementation, it is essential to establish population specific reference datasets, validate predictive models under local genetic contexts, and enforce standardized protocols that ensure data protection and regulatory compliance within judicial frameworks [84]. In this context, informed consent must play a central role by explicitly detailing the potential forensic uses of genetic data, its retention period, and restrictions on secondary use [85,86]. Participants should be informed that their data may contribute to population databases or forensic validation studies, but not to investigative databases without renewed authorization.

## 6. Discussion: Future Directions and Recommendations

As FDP continues to evolve, its successful and responsible implementation in Latin America will depend on targeted scientific, institutional, and ethical advances [16]. Given the region’s rich genetic diversity and unique forensic challenges, future efforts must prioritize the development of locally relevant tools and the strengthening of technical and human capacities [60].

A critical step forward is the validation of existing prediction models in Latin American populations. Most current FDP systems, such as HIrisPlex-S, were designed based on European datasets, limiting their predictive reliability in populations with high levels of admixture [65]. To improve accuracy, these models must be rigorously tested on Latin American samples that reflect the wide range of ancestral combinations found across the region [87]. Only through systematic validation can we assess which phenotypes are reliably predicted and where adjustments are necessary [88].

In parallel, there is a pressing need for the development of population-specific algorithms. Rather than relying solely on imported tools, regional research institutions and forensic laboratories should collaborate to design predictive models tailored to local genetic profiles [89]. This involves identifying additional SNVs or gene variants that may be informative in mixed ancestry contexts, as well as integrating ancestry-informative markers to improve the interpretability of phenotypic outputs [15]. Locally adapted models will not only enhance accuracy but also increase public trust in the fairness and cultural relevance of FDP [65]. Recent studies have emphasized the importance of regional reference data or representative biobanks for improving the accuracy of FDP. Becher et al. (2024) [90] characterized the genetic landscape of FDP markers among Mediterranean populations, revealing significant allele frequency differences in loci such as *TCHH*, *PRKCE*, *OCA2*, *MC1R*, and *MFSD12* when compared to other global groups. Their findings underscore how population-specific genetic variation can influence the predictive reliability of externally visible traits. This regionalized approach supports similar initiatives in Latin America, where the high degree of genetic admixture also demands the development of local databases and validation studies to ensure accurate and equitable phenotype prediction in forensic applications [90].

The establishment of representative biobanks is essential for advancing genetic and forensic research in Latin America. These facilities collect and store genetic and phenotypic data from diverse populations in a structured, transparent, and ethically compliant manner [91]. Ethical compliance includes obtaining informed consent from participants, ensuring data anonymization, and restricting data access to authorized personnel, in accordance with national and international regulations for human genetic research [81,91]. Regional biobanks can serve as reference datasets for model training and validation, providing the empirical foundation for population-specific prediction algorithms [92]. These frameworks must ensure robust data protection, interoperability, and equitable benefit-sharing to maintain transparency and public trust [93]. To enhance predictive accuracy in admixed populations, models should integrate locally informative SNVs, ancestry-informative markers, and cross-population validation. Implementation should proceed through a phased approach, beginning with pilot datasets from national reference laboratories and progressively expanding through multicenter collaborations across Latin America.

While population scale biobanks and high throughput genotyping are essential, the predictive validity of FDP in Latin America depends on accurately modeling the region’s intricate admixture. Differences in allele frequencies, linkage disequilibrium, and epistatic relationships among biogeographic ancestries suggest that the same phenotype may be best predicted by different marker panels in various ancestry strata. Thus, ancestry-aware feature selection, interaction screening, and the assessment of supplementary variants within canonical loci (*HERC2*/*OCA2*, *SLC24A5*, *SLC45A2*, *MC1R*), alongside potential modifier genes in Latin American cohorts, will improve accuracy and equity in phenotype prediction for admixed populations facilitating analytical/external validation [18,39,43,94,95].

Finally, to ensure responsible application, it is essential to train forensic professionals in the interpretation and communication of phenotypic predictions [96]. FDP outputs are probabilistic and context-dependent; therefore, professionals must be equipped not only with the technical skills to interpret prediction reports but also with the communication tools to explain limitations, degrees of certainty, and ethical implications to legal authorities, families of victims, and the public. Interdisciplinary training including genetics, bioethics, law, and communication will be vital to bridging science and practice in a region where social sensitivities and historical injustices must be carefully considered [57]. The future of FDP in Latin America lies in a balanced approach that combines scientific innovation with ethical responsibility and institutional capacity-building.

## 7. Conclusions

The prediction of eye, hair, and skin color from DNA represents a promising forensic tool with growing applicability in human identification. Although several challenges persist in Latin American populations due to their high genetic diversity, progress can be achieved through the establishment of regional biobanks, local validation of predictive models, and the integration of interdisciplinary training programs that combine genetics, bioinformatics, and legal expertise. Furthermore, the development of clear ethical and legal frameworks is essential to ensure the responsible and equitable use of forensic DNA phenotyping. Understanding the complex genetic architecture underlying pigmentation driven by major and minor genes, specific SNVs with measurable phenotypic effects, and polygenic mechanisms remains a cornerstone for advancing both anthropological and forensic research focused on externally visible traits. Future research should prioritize large-scale multiethnic GWASs and the creation of region-specific predictive panels to enhance model accuracy and applicability in admixed Latin American populations.

## Data Availability

No new data were created or analyzed in this study. Data sharing is not applicable to this article.
